# Influence of Short-Term Consumption of *Hericium erinaceus* on Serum Biochemical Markers and the Changes of the Gut Microbiota: A Pilot Study

**DOI:** 10.3390/nu13031008

**Published:** 2021-03-21

**Authors:** Xiao-Qian Xie, Yan Geng, Qijie Guan, Yilin Ren, Lin Guo, Qiqi Lv, Zhen-Ming Lu, Jin-Song Shi, Zheng-Hong Xu

**Affiliations:** 1 School of Pharmaceutical Sciences, Jiangnan University, Wuxi 214122, China; 6181504006@stu.jiangnan.edu.cn (X.-Q.X.); renyilin@jiangnan.edu.cn (Y.R.); 1162190115@stu.jiangnan.edu.cn (Q.L.); shijs@163.com (J.-S.S.); 2 National Engineering Laboratory for Cereal Fermentation Technology, Jiangnan University, Wuxi 214122, China; qijie.guan@jiangnan.edu.cn (Q.G.); 7170201007@stu.jiangnan.edu.cn (L.G.); zmlu@jiangnan.edu.cn (Z.-M.L.); zhenghxu@jiangnan.edu.cn (Z.-H.X.); 3 Jiangsu Engineering Research Center for Bioactive Products Processing Technology, Jiangnan University, Wuxi 214122, China; 4 Key Laboratory of Industrial Biotechnology of Ministry of Education, School of Biotechnology, Jiangnan University, Wuxi 214122, China

**Keywords:** medicinal mushroom, gut microbiota, *Faecalibacterium prausnitzii*, *Kineothrix alysoides*, kidney stones, dietary regulation, *Hericium erinaceus*, gout

## Abstract

*Hericium erinaceus* (*H. erinaceus*) is widely studied as a medicinal and edible fungus. Recent studies have shown that *H. erinaceus* has protective effects for diseases, such as inflammatory bowel disease and cancer, which are related to gut microbiota. To investigate the benefits of *H. erinaceus* intake on gut microbiota and blood indices in adulthood, we recruited 13 healthy adults to consume *H. erinaceus* powder as a dietary supplement. Blood changes due to *H. erinaceus* consumption were determined by routine hematological examination and characterized by serum biochemical markers. Microbiota composition was profiled by 16S ribosomal RNA gene sequencing. Results showed that daily *H. erinaceus* supplementation increased the alpha diversity within the gut microbiota community, upregulated the relative abundance of some short-chain fatty acid (SCFA) producing bacteria (*Kineothrix alysoides*, *Gemmiger formicilis*, *Fusicatenibacter saccharivorans*, *Eubacterium rectale*, *Faecalibacterium prausnitzii*), and downregulated some pathobionts (*Streptococcus thermophilus*, *Bacteroides caccae*, *Romboutsia timonensis*). Changes within the gut microbiota were correlated with blood chemical indices including alkaline phosphatase (ALP), low-density lipoprotein (LDL), uric acid (UA), and creatinine (CREA). Thus, we found that the gut microbiota alterations may be part of physiological adaptations to a seven-day *H. erinaceus* supplementation, potentially influencing beneficial health effects.

## 1. Introduction

Intestinal health includes a balanced gut microbiota, effective immune regulation, and optimal nutrient utilization or absorption [1]. Disrupting gut microbiota homeostasis has a significant impact on human health. Other than environmental stimuli, diet acts as a critical modulator of the gut microbiota, influencing both composition and function of the community [2]. Specific carbohydrates can differentially promote the growth of distinct gut microbes, with metabolites released during the dynamic adjustment of the microbial ecosystem influencing their host. For example, some studies have shown that an alteration of the gut microbiota is associated with diet, and plays an important role in human health and metabolic diseases [3,4]. Obesity-related diseases, such as metabolic syndrome, type 2 diabetes, cardiovascular disease, and inflammatory bowel diseases, have also been associated with changes in the gut microbiome composition, with nutrient-gut microbiota interactions acting as possible therapeutic targets [5,6]. Another study proposed that the etiology of brain disorders may be partially due to the gut microbiota [7]. Phenylacetylglutamine, a gut microbiota-derived metabolite, is associated with cardiovascular disease in humans [8].

*H. erinaceus*, also known as Yamabushitake mushroom, is a kind of white and fleshy fungus growing primarily on dead or dying wood. It belongs to *Hericiaceae*, *Hericiales*, *Homobasidiomycetes*, *Hymenomycetes*, and *Basidiomycota*. Many studies have focused on the benefits of *H. erinaceus* powder on human health. H. *erinaceus* may have therapeutic potential against human leukemia since it activates mitochondria-mediated caspase-3 and caspase-9 apoptosis [9]. In the brain, studies have found that *H. erinaceus* powder improves mild cognitive impairment [10]. Additionally, *H. erinaceus* improves depression and anxiety-related mood disorders and nocturnal rest via modulation of brain-derived neurotrophic factor (BDNF) and its precursor pro-BDNF in serum [11]. Furthermore, the fungal proteins from *H. erinaceus* can promote the antitumor efficacy of fluorouracil (5-Fu) by altering microbiota composition, improving immune-inflammatory response, and restoring homeostasis [12].

Both primary macronutrients and numerous micronutrients modify the gut microbiota. Carbohydrates play an important role in this process, as gut microbes possess carbohydrate-degrading enzymes to use indigestible carbohydrates as their primary energy source. As diet-responsive members of the microbiota often represent a small proportion of the total community, this requires a better understanding of whether changes in populations are sufficient to elicit physiological outcomes in the host [13]. Without extreme external pressure, the gut microbiota of healthy adults can be characterized as relatively stable, both in terms of resistance and resilience [14]. This considerable resilience allows it to return to its original state when challenges cease, resulting in a negligible effect [15]. As *H. erinaceus* plays a role in protecting human health, we performed a preliminary study in healthy young adults to investigate the changes of gut microbiota responding to *H. erinaceus* intervention and explored the association of blood biochemical indices with gut microbiota.

## 2. Materials and Methods

### 2.1. Subjects

The dry powder of *H. erinaceus* in submerged culture was obtained from Jiangsu Shenhua Pharmaceutical Co., Ltd., China (Batch No: 20180712). Components of *H. erinaceus* powder include polysaccharides, peptides, crude fat, and some trace elements [16]. Supplementary information (Tables S1 and S2) provides additional details.

This study was conducted between November 2018 and December 2018 in Wuxi, China. All participants were informed of the procedure at the recruitment time and were asked to sign an informed consent form before the study was conducted. This study was approved by the Medical Ethics Committee of Wuxi Second People’s Hospital. A total of 13 healthy people (7 females and 6 males), with a mean age of 30.0 years old (standard deviation 4.9), participated in this research. Blood sampling for biochemical indicators, routine hematological examinations, and feces sampling for gut microbiota analyses were conducted according to the experimental design. Subjects with BMIs (kg/m^2^) ranging from 19.6 to 23.4 were enrolled in this study. Participant exclusion criteria included any antibiotic use during the previous one month prior to the study, a history of any gastrointestinal disease, following a vegetarian diet, pregnancy or lactating at the time of participation, and actively trying to lose or gain body weight. Participants were instructed to stop drinking, smoking, and using any probiotics, prebiotics, or other dietary supplements two weeks before the study and during the study.

### 2.2. Study Design

**Figure 1:**
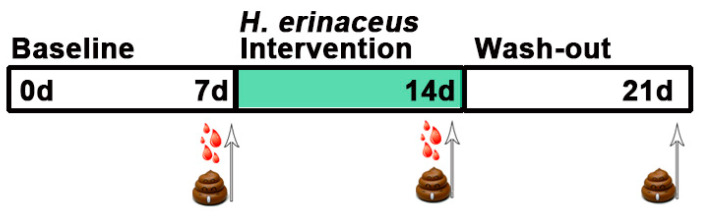
Brief timeline of the study design and sampling. Each of the three phases of the experiment was maintained for a week as follows: baseline, a phase to balance some stimulus which may affect the samples; intervention, a period to consume *H. erinaceus* powder with the daily diet; and wash-out, the final stage to remove *H. erinaceus* powder from the daily diet and maintain a regular diet. Samples were taken prior to the start of the next phase.

The experiment consisted of three parts. The first part is a seven-day baseline period where all participants maintained a regular diet. This was followed by a seven-day intervention period, when all participants were instructed to take 1 g of *H. erinaceus* powder three times a day. Following the intervention period, a seven-day wash-out period was scheduled. Participants were required to have fecal samples collected during these three periods. Blood samples were collected at the end of the baseline and intervention period. Specifically, the fecal samples were collected at home with individually packaged sterile stool tubes and stored at −20 °C temporarily. These fecal samples were then transported to the laboratory for storage at −80 °C within 12 h. All fecal samples were individually sealed and stored. The blood samples were drawn after an overnight fast (12 h) at the end of the seven-day baseline and the seven-day intervention period. A simplified illustration is shown in Figure 1.

### 2.3. Assessment of Samples

Total genomic DNA from samples was extracted using a Soil DNA Kit according to the manufacturer’s instructions [17]. To investigate the impact of *H. erinaceus* on the gut microbiomes of healthy humans, we analyzed the composition and abundance of gut microbiomes by next generation sequencing of the V3 and V4 regions of 16S rRNA genes of the bacteria in feces. The V3 and V4 regions were amplified using forward primers containing the sequence ‘CCT ACG GRR BGC ASC AGK VRV GAA T’ and reverse primers containing the sequence ‘GGA CTA CNV GGG TWT CTA ATC C’. All samples were sequenced using the Illumina MiSeq platform. DADA2, an R package, which models and corrects Illumina-sequenced amplicon errors, was used to identify sequences. DADA2 infers sample sequences exactly and identifies differences of as little as one nucleotide [18]. The quality filtering method of sequences was as follows: only reads with quality value scores of ≥20 for more than 99% of the sequence were extracted for further analyses. Silva_species_assignment_v138 reference database was used to assign taxonomy. The 16S amplicon sequencing variants result (ASVs) was processed using a comprehensive visual microbiome data analysis web tool (Microbiome Analyst) [19]. Spearman correlation analysis was produced using the “corrplot” function with R package corrplot v0.84 as previously described [20]. EdgeR package was used to identify the differentially enriched ASVs, which utilized relative log expression as a default normalization and assumes a negative binomial model for count distributions [21]. Features are considered to be significant based on their adjusted *p*-value (<0.05). 

We collected blood samples from each subject with both EDTA-K2 anticoagulation tubes and conventional blood collection tubes. The samples were centrifuged at 1200× *g* for 10 min, and the supernatants were collected. The supernatants were stored at 4 °C before being transferred to the laboratory. All analytical tests were completed prior to 24 h post-collection. An AU680 automatic analyzer (Beckman Coulter, Brea, CA, USA) was used to determine all biochemical analyses.

### 2.4. Statistical Analysis

The fecal samples were grouped according to the collection period, and the following analyses were performed accordingly. Comparisons between groups were performed using the nonparametric Kolmogorov-Smirnov test and blood samples were analyzed with a parametric *t*-test. Data analysis was processed by GraphPad Prism 8.2 software (version 8.2.1 Windows version, GraphPad Software, San Diego, USA).

## 3. Results

### 3.1. Effect of H. erinaceus Powder on the Composition of Gut Microbiota

Analysis of the demultiplexed paired-end reads generated a total of 6,863,390 reads, which ranged from 62,243 to 115,031, with an average of 92,748 reads per sample. After denoising and removing chimeras using the DADA2 pipeline, we constructed a higher resolution ASV table with a total of 2,502,113 reads ranging from 15,858 to 43,989 (Supplementary Data Matrix 1). Taxonomic classification using the Silva_species_assignment_v138 reference database identified 5966 ASVs (Supplementary Data Matrix 2). We normalized ASVs counts for the participants, and a rarefaction curve was generated at a depth of 7500 (Figure S1). Adequate depth coverage was reached as determined by the individual curves plateauing out on the rarefaction curve.

**Figure 2:**
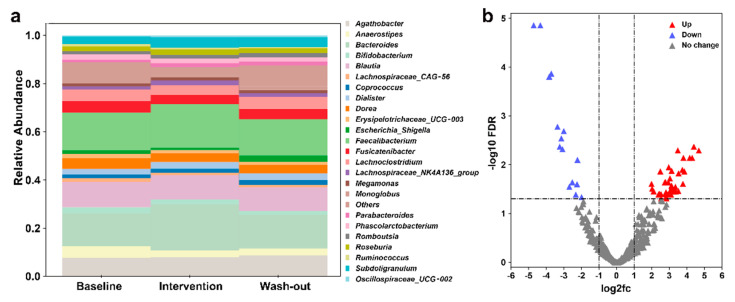
Effects of *H. erinaceus* intervention on gut microbes. (**a**) A chart showing the 25 most abundant bacterial genera (relative abundance). (**b**) Gut microbiomes with *H. erinaceus* intervention are enriched and depleted for certain amplicon sequencing variants (ASVs) compared to baseline. The red points are the ASVs that are significantly upregulated, the purple points are the ASVs that are significantly downregulated, and the gray points shows ASVs with no significant changes.

Filtered data, which has low quality and/or uninformative features removed, were used to show the taxonomic composition using a stacked bar plot. Phylum to family-level classification of bacteria identified in fecal samples are shown in Figure S2. Results of the 25 most abundant bacterial genera classifications (relative abundance) are shown in Figure 2a. We observed an increased relative abundance of some beneficial bacteria, such as *Bifidobacterium* and *Bacteroides*, after short-term *H. erinaceus* supplementation. The relative abundance of SCFAs-producing bacteria such as *Roseburia* and *Faecalibacterium* also increased. 

To further explore the influence of *H. erinaceus* on gut microbiota, we constructed a volcano plot to show the most different 500 ASVs and found that a total of 50 ASVs which were significantly changed (Figure 2b). Specific data is shown in Supplementary Data Matrix 3. We then found that *H. erinaceus* diet intervention upregulated the relative abundance of some probiotics species that can produce SCFAs (*Roseburia faecis*, *Faecalibacterium prausnitzii*, *Eubacterium rectale*, *Fusicatenibacter saccharivorans*, *Kineothrix alysoides*, *Gemmiger formicilis*, *Dorea longicatena*), and downregulated the relative abundance of some pathobionts (*Streptococcus thermophilus*, *Roseburia intestinalis*, *Bacteroides caccae*, *Bacteroides caccae*, *Anaerostipes hadrus*).

### 3.2. Effect of H. erinaceus Powder on the Diversity of Gut Microbiota

**Figure 3:**
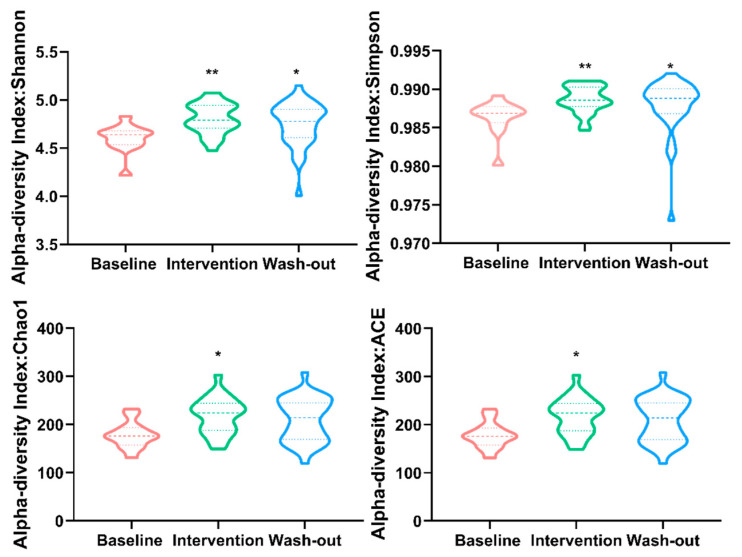
Alpha diversity measure using Chao1, ACE, Simpson, and Shannon at ASV level represented as violin plots. Each plot represents the diversity distribution of a group present (Data use nonparametric statistics Kolmogorov-Smirnov test for significant difference analysis, statistical significance: * *p*-value < 0.05, and ** *p*-value < 0.01 compared with baseline group).

Alpha diversity can be characterized via the total number of species (richness), the abundances of the species (evenness), or measures that considered both richness and evenness. Our alpha diversity analysis was performed using the phyloseq package. The results are plotted in Figure 3 across samples and reviewed as violin plots for each period. To determine whether alpha diversity differs across the three periods, ASVs were calculated without filtration as recommend [22]. Alpha diversity estimates including Chao1, ACE, Simpson, and Shannon indices of gut microbiota in the *H. erinaceus* intervention period were significantly increased compared to baseline phase. The Simpson and Shannon indices also increased during the wash-out period. These results indicate that *H. erinaceus* powder supplementation could significantly increase both richness and evenness of human gut microbiota, and may produce a slight retention effect when stopped.

Analysis beta diversity, using *H. erinaceus* powder consumption or control as the index, showed no statistically significant differences between groups by principal coordinates analysis (PCoA) (Figure S3). Beta diversity comparison using Bray-Curtis distance showed differences in community composition in all participants as illustrated in the unrooted graph (Figure S4). These results indicate that the community compositions of individuals are separate.

### 3.3. Effect of H. erinaceus Powder on Blood Parameters related to Metabolic Diseases

**Figure 4:**
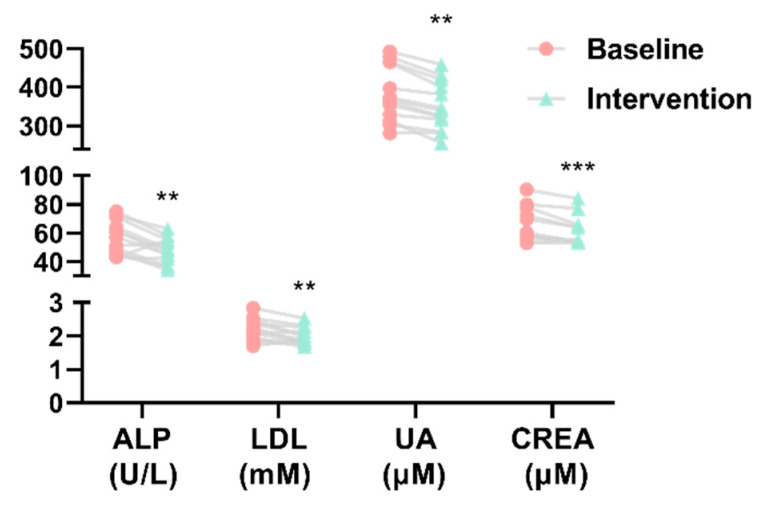
Serum concentration of blood biochemical indices (** *p*-value < 0.01, *** *p*-value < 0.001 compared with baseline group). ALP (alkaline phosphatase), LDL (low-density lipoprotein), UA (uric acid), and CREA (creatinine).

Compared to baseline period, ALP, LDL, UA, and CREA levels tend to be lower after *H. erinaceus* intervention (Figure 4), while other hepatocyte-specific enzymes, such as aspartate aminotransferase and alanine aminotransferase, showed no significant differences with *H. erinaceus* intervention (Figure S5). Additionally, routine hematological examinations of whole blood showed that consuming *H. erinaceus* powder did not affect the composition of blood cells (Table 1).

### 3.4. Correlation between Gut Microbiota and Blood Biochemical Indices

**Figure 5:**
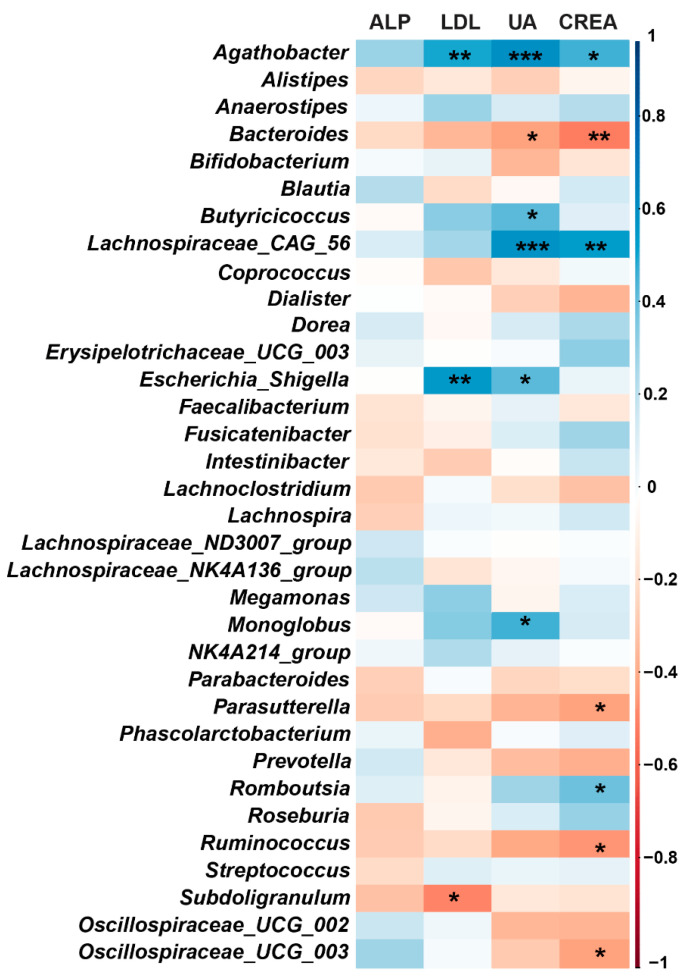
Correlation between gut microbiota at the genus level and serum biochemical indices. Heatmap representing color-coded Spearman’s correlations of the indices. Blue indicates a positive correlation whereas red indicates a negative correlation. The deeper the color, the stronger the positive or negative correlation. * *p* < 0.05, ** *p*< 0.01, *** *p* < 0.001.

Spearman correlation analysis between gut microbiota changes at the genus level and serum biochemical indices are listed in Figure 5. We found that *Agathobacter* and *Escherichia_Shigella* were significantly positively correlated with LDL and UA in serum; *Bacteroides*, *Parasutterella*, *Ruminococcus*, and *Oscillospiraceae_UCG-003* were significantly negatively correlated with CREA in serum. The potential beneficial genus *Bacteroides*, *Parasutterella*, *Ruminococcus,* and *Subdoligranulum* were increased after *H. erinaceus* supplementation, while some harmful genera such as *Escherichia_Shigella* were decreased (Figure 2a). These results indicate that changes in gut microbiota may contribute to the effect of *H. erinaceus* on some of the serum biochemical indices.

## 4. Discussion

We aimed to investigate alterations in gut microbiota associated with short-term supplementation of *H. erinaceus*. We found that some beneficial bacteria were significantly increased, but some pathobionts related to metabolic arthritis (gout) and tumor immune escape significantly decreased in the feces of the participants. Also, we identified a significant increase in alpha diversity of the microbiota community after *H. erinaceus* supplementation. To our surprise, the levels of serum biochemical indices, including ALP, LDL, UA, and CREA, were markedly decreased after *H. erinaceus* intervention. We further identified which gut microbes were positively or negatively correlated with ALP, LDL, UA, and CREA levels in serum.

The most prevalent limitation of our study is its small sample size. However, the data obtained still allow us to better understand the role of *H. erinaceus* in human gut microbiota. There have been many studies on the probiotic effect of *H. erinaceus* in animals. For example, *H. erinaceus* extracts ameliorated inflammatory bowel disease in rats and significantly changed the gut microbiota structure [23]. Another study showed that the protein isolated from *H. erinaceus* could play a probiotic role by regulating the composition of gut microbiota and improving immune system function in mice [24]. The polysaccharides of *H. erinaceus* can alleviate colitis by modulating the composition of intestinal microbes in rodents [25,26], and can repair Muscovy duck reovirus associated injures of small intestinal mucosal immunity in Muscovy ducklings [27]. Besides these health effects, clinical research has shown that *H. erinaceus* has a particular therapeutic effect on the treatment of depressive disorder [28]. Gut microbes may play an important role in mental health through the gut-brain axis [29].

Although dietary shifts have the potential to modify the composition and functions of gut microbiota within the course of days, the exact time frame might be person-specific [30]. Our study designed a seven-day intervention experiment that added *H. erinaceus* powder to the daily food. We performed 16S rRNA gene sequencing and found that consumption of *H. erinaceus* powder could increase the diversity of gut microbiota. However, it did not significantly disrupt the balance of the gut microbiota, and the alterations could be gradually restored without *H. erinaceus* consumption. A large number of studies have shown that the diversity of gut microbes is closely related to human health, and the decline in gut microbial alpha diversity is associated with the increased prevalence of common metabolic diseases [31]. For example, the diversity of gut microbes decreased in patients with bipolar disorder and the number of butyrate-producing bacteria was significantly reduced compared with healthy people [32].

In terms of the composition of gut microbiota, we found some significant changes after *H. erinaceus* supplementation at the species level. For example, the relative abundance of the bacteria *Bifidobacterium longum*, *Parabacteroides merdae*, and *Anaerostipes hadrus*, which promote tumor immunotherapy response, were significantly increased after consumption of *H. erinaceus*. Additionally, the bacteria *Roseburia intestinalis*, which is associated with not responding to tumor immunotherapy, was downregulated [33,34]. Also, *H. erinaceus* supplementation could reverse the relative abundance of certain gut bacteria enriched or reduced in some diseases, such as *Bacteroides caccae* enriched in metabolic arthritis patients [35], *Phascolarctobacterium* enriched in kidney stones patients [36], *Romboutsia timonensis* enriched in patients with the chronic obstructive pulmonary disease [20], *Streptococcus thermophilus* that is a predictor of poor prognosis for liver cirrhosis [37], and *Coprococcus comes* decreased in patients with chronic widespread pain [38]. Studies have shown that some bacteria which can contribute to human health include SCFAs-producing bacteria (*Faecalibacterium*, *Bacteroides*, *Subdoligranulum*) and other probiotics like *Parabacteroides*, *Dialister* [39]. A previous study found that the abundance of *Faecalibacterium*, *Megamonas*, and *Roseburia* in patients with metabolic arthritis were significantly decreased [18]. Our results, measured using biochemical blood indices, demonstrate a downregulation in UA and CREA. Therefore, we propose that alterations in gut microbiota due to *H. erinaceus* intervention may be related to metabolic arthritis. In addition to the upregulation of SCFAs-producing bacteria after *H. erinaceus* intervention, we also found an increased abundance of *Parabacteroides*, which degrade fiber, pectin, and regulate glycolipid metabolism [40].

Regarding the results of blood indices, ALP, LDL, UA, and CREA levels were significantly decreased after *H. erinaceus* intervention in our study. Studies have shown that increases in ALP are related to the poor prognosis of primary biliary cirrhosis [41]. Increased intestinal alkaline phosphatase activity indicates that there are many potential pathogenic bacteria in the intestine [42]. Obstruction or damage to the liver can also lead to increased ALP levels [43]. Interestingly, cardiovascular diseases are related to dyslipidemia and findings suggest that the gut microbiota interferes with the lipid metabolism of the host. Secondary bile acids produced by the gut microbiota participate in liver metabolism and lipid regulation. Low LDL levels can reduce the risk of cardiovascular disease associated with elevated lipoproteins [44]. Elevated levels of uric acid in serum or urine are closely related to metabolic arthritis, kidney, and vascular diseases. In type 1 diabetes, a higher UA level is associated with a higher risk of decline in kidney function and mortality [45]. CREA, existing in blood and urine, is the key biomarker for assessing chronic kidney disease and monitoring its progress in clinical medicine [46]. In the rat model of chronic kidney disease, increases in *Eggerthellalenta* and *Fusobacterium nucleatum* in the gut microbiota increased the production of urinary toxins and accelerated the progression of kidney disease. However, the beneficial bacteria *Bifidobacterium* can reduce the abundance of these two bacteria, thereby reducing the level of toxins and severity of kidney disease [47].

Changes in gut microbes are dynamic, and the factors that influence the gut microbiota are numerous. We investigated gut microbial changes due to *H. erinaceus* intervention to find out more details associated with the alterations in the serum biochemical indices. Further studies should consider expanding the sample population size and extending the intervention period, as there are significant individual differences in the composition of gut microbiota.

## 5. Conclusions

Overall, we supplemented *H. erinaceus* powder into healthy people’s diets to investigate its effect on both gut microbiota and serum biochemical indices. This small pilot study revealed that *H. erinaceus* powder, as a supplementary food, can regulate the composition of gut microbiota, and that changes of the gut microbiota are correlated with some biochemical indicators in the blood.

## Figures and Tables

**Table 1:** Routine hematological examinations of whole blood for baseline and intervention period.

Blood Routine Index	Baseline (*n* = 13)	Intervention (*n* = 13)	*p*-Value
Red blood cell (10^9^/L)	4.848 ± 0.311	4.802 ± 0.316	0.077
Leukocyte (10^9^/L)	6.522 ± 0.923	6.495 ± 1.106	0.883
Hemoglobin (g/L)	144.333 ± 11.448	143.250 ± 11.959	0.286
Platelet (10^9^/L)	247.308 ± 35.569	251.231 ± 54.104	0.564
Lymphocyte (10^9^/L)	2.465 ± 0.576	2.244 ± 0.417	0.052
Monocytes (10^9^/L)	0.382 ± 0.061	0.389 ± 0.061	0.615
Neutrophil (10^9^/L)	3.508 ± 0.613	3.705 ± 0.892	0.106
Eosinophil (10^9^/L)	0.150 ± 0.114	0.143 ± 0.096	0.590

## Data Availability

The Study is managed by the National Engineering Laboratory for Cereal Fermentation Technology, Jiangnan University. For data access, please contact the Xu Group at gengyan@jiangnan.edu.cn.

## Supplementary Materials

The following are available online at https://www.mdpi.com/2072-6643/13/3/1008/s1, Figure S1: Rarefaction curve using original dataset, as the sequencing depth increased up to 5000, no new sequence was generated, indicating that the sequences generated by the samples were sufficiently covered. Red line: Baseline group; green line: Intervention group; blue line: Wash-out group, Figure S2: Taxonomic composition of the community at a different level using stacked bar plot. Taxonomic relative abundance is viewed at a sample-group level based on *H. erinaceus* intervention; y-axis represent relative abundance of bacterial, Figure S3: Microbial changes between groups showing in β diversity PCoA diagram calculated by the distance between groups (*p*-value < 0.815), each axis reflects the percent of the variation between the samples with the X-axis representing the highest dimension of variation and the Y-axis representing the second-highest dimension of variation. Samples displayed on PCoA plots are colored based on *H. erinaceus* intervention grouping, genus abundance levels were measures, Figure S4: Unrooted graph shows the result of clustering analysis of all samples with Bray-Curtis distance to clustering to minimize the sum of squares of any two clusters, Figure S5: Serum concentration of blood biochemical indexes from baseline period and *H. erinaceus* intervention; alanine aminotransferase (ALT), aspartate aminotransferase (AST), glutamyl transferase (GGT), triglyceride (TG), blood urea nitrogen (BUN), cholesterol (CHOL), Table S1: Chemical compositions of *H. erinaceus*, Table S2: Trace element of *H. erinaceus*, Supplementary Data Matrix 1: Amplicon sequence variant table after denoised with DADA2 pipeline, Supplementary Data Matrix 2: Taxa for classification of bacteria assigning with Silva_species_assignment_v138 database, Supplementary Data Matrix 3: The difference matrix of the ASV defined by the volcano map.
